# Kidney Stones in Several Spinal Abnormalities: A Challenging Treatment

**DOI:** 10.1089/cren.2015.0043

**Published:** 2016-01-01

**Authors:** Maximiliano Lopez Silva, Horacio Sanguinetti, Santiago Battiston, Patricio Alvarez, Norberto Bernardo

**Affiliations:** ^1^ Urology Department, Hospital de Clínicas José de San Martín, Buenos Aires, Argentina.; ^2^ Urology Department, Clínica San Camilo, Buenos Aires, Argentina.

## Abstract

Patients with severe skeletal deformities are a challenging group to treat. A female, white, 35-year-old presented with right kidney stones located in renal pelvis, lower calyx, and upper ureter. She was affected by severe spinal deformity with restrictive respiratory obstruction, caused by kyphoscoliosis. Percutaneous nephrolithotomy in supine position was performed, achieving complete removal of kidney stones. The treatment of renal stones in this patient was complex, so special attention to respiratory function was mandatory; this was a challenging but feasible situation.

## Introduction and Background

Percutaneous nephrolithotomy (PCNL) has become the first-line treatment option for complex, large, and staghorn calculi, since its advent in the 1970s. At present, due to the development of PCNL, surgery has become a rarely used procedure.

Spinal deformities and another abnormal body habitus constitute a challenge during the surgical procedure and for anesthetic management. Right handling of urolithiasis in this group of patients may be difficult because of anatomic variations and respiratory dysfunction, and stone size may not be the only factor in deciding the best treatment.

Our aim is to highlight the difficulties that can occur in these cases, which can be effectively treated.

## Presentation of Case

The patient is a female, white, and 35 years old. Surgical history: kyphoscoliosis correction at age 3 (without reaching the expected results). Medical history: restrictive pulmonary disease. She consulted in June 2015 with 6 months of right lumbar pain, with flank irradiation, and no associated fever.

**FIG. 1.**
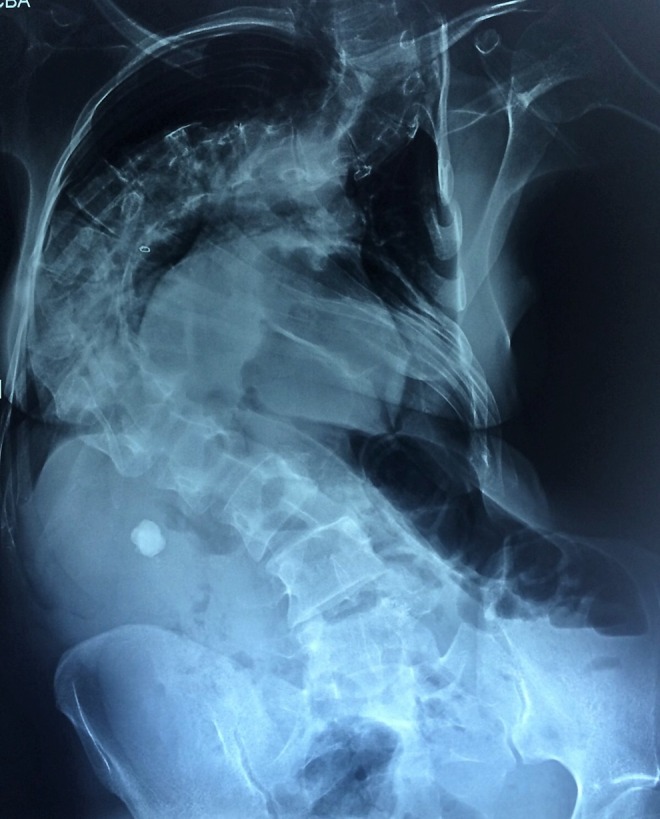
KUB with right renal pelvis stone.

**FIG. 2.**
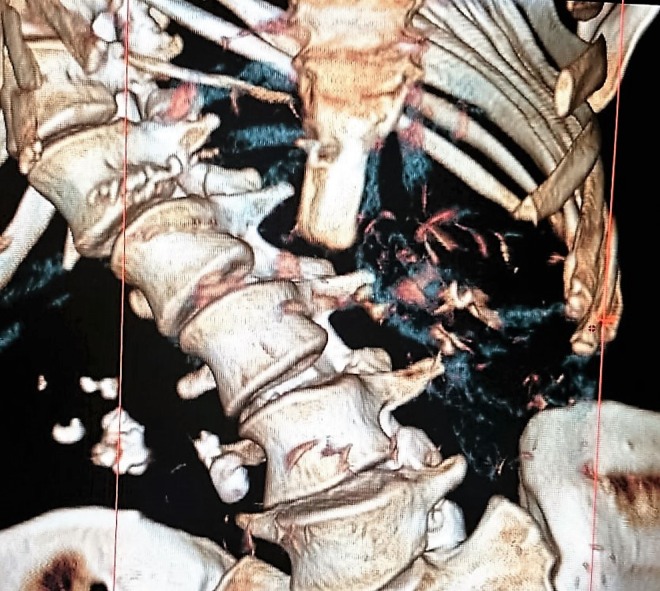
Abdominal CT that shows right stones and severe kyphoscoliosis (reconstruction, front vision). CT, computed tomography.

**FIG. 3.**
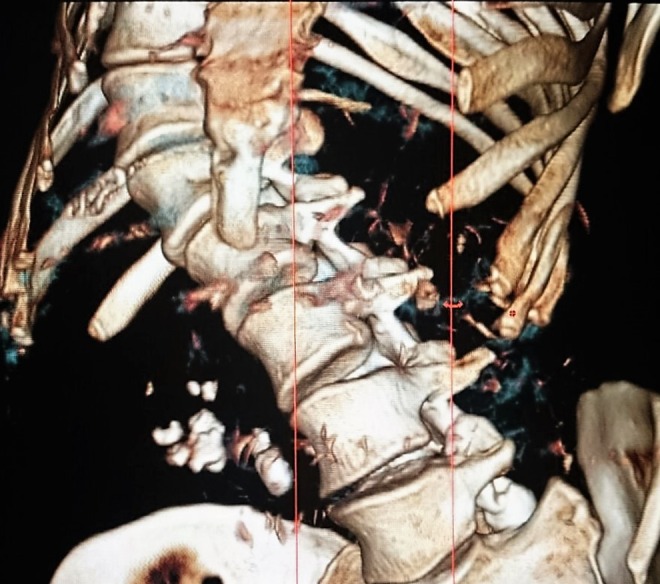
Abdominal CT that shows right stones and severe kyphoscoliosis (reconstruction, lateral vision).

KUB: stones were located in right renal pelvis (Fig. 1). Extracorporeal shockwave lithotripsy was performed. After that, ultrasonography showed two stones in the right renal pelvis (15 and 8 mm size) and computed tomography (CT) showed a 17-mm stone in the right renal pelvis, 7-mm stone in the lower calix, and 10-mm stone in the proximal ureter; it also reported severe associated kyphoscoliosis (Figs. 2 and 3).

Urine culture was negative and laboratory tests showed normal parameters.

Lung function test evidenced restrictive pattern.

The patient was placed in the modified lithotomy position. Cystoscopy was performed using a 20F cystoscope (Karl Storz-Endoskope^®^, Tuttlingen, Germany); retrograde urography with fluoroscopic guidance confirmed the presence of renal pelvic, lower calyceal, and proximal ureteral stones. A 0.035′′ hydrophilic guide (Cook Medical Devices^®^, Bloomington, IN) was located.

In a second time, PCNL in modified supine position was performed. This position was chosen because of the anatomical restriction of the patient to get another position.

Access to the urinary tract was achieved in the lower calix by the triangulation technique with fluoroscopy guidance. An 18-gauge needle was used to reach the renal cavities. Dilatation of the tract was performed using a dilatation balloon UltraxxTM (Cook Medical Devices); opaque 30F Amplatz sheath (Cook Medical Devices) was utilized. During the procedure, 26F Nephroscope (Karl Storz-Endoskope) and StoneBreakerTM (Cook Medical Devices) for lithotripsy were used, removing fragments with trident forceps (Karl Storz-Endoskope) and NCircle^®^ Perc Stone extractor device (Cook Medical Devices). Absence of residual stones was corroborated by direct and fluoroscopic vision.

**FIG. 4.**
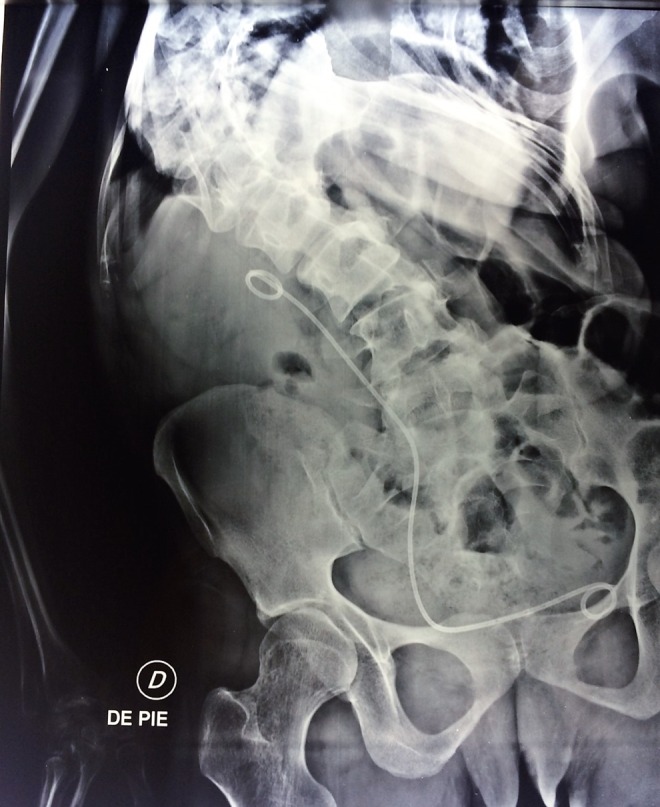
Postsurgical KUB.

The procedure was completed with the placement of a Double-J ureteral catheter 6F-26 cm (Cook Medical Devices) and 20F Foley catheter as nephrostomy tube. Postsurgical KUB is shown in Figure 4.

A week after the procedure, Double-J stent was removed without complications.

## Discussion and Literature Review

Spinal deformity (scoliosis, kyphosis, or kyphoscoliosis) is caused by the pathologic curvature of the thoracic and/or lumbar regions of the spine. It is estimated that scoliosis affects 2% of women and 0.5% of men in the general population, although rates may differ quite significantly based on the specific definition of scoliosis and which patient population is being studied.^1^

The treatment of urolithiasis in patients with abnormal body habitus, such as morbidly obese or spinal cord injury patients, presents severe, unique challenges for the urologist because of technical difficulties that reduce the stone-free rate and a higher rate of complications, often necessitating creativity and innovation.^1^ Patients with severe kyphosis cannot be placed in the prone position, because their respiratory restrictive function is worsened by the secondary restriction of diaphragm movement by the increased intraabdominal pressure.^2^

The lower stone-free rate is shown in a study that classifies any stone in patients with spinal abnormalities as Guy's stone score grade IV, this grade being the least possibility of reaching stone-free rate.^3^

Some authors, as Goumas-Kartalas et al., consider patients with spinal deformities and urolithiasis who are undergoing PCNL as a special category, because even if there are some common risks and issues with other comorbidities such as obesity or paraplegia, these patients present unique and extreme anatomic variations that necessitate a different approach in planning and performing percutaneous procedures.^1^

In the approach to PCNL in patients with spinal deformity, three major issues should be addressed: (1) Anesthesiologist and cardiorespiratory issues; (2) patient position on the operating table, and (3) percutaneous access modality.

Regarding the first point, anesthesia-related difficulties depend on the type and severity of skeletal deformities and may be exacerbated by the use of the prone position during PCNL. Restrictive lung disease should be taken into consideration. Also, these deformities may cause difficulty in tracheal intubation. Owing to all these reasons, regional anesthesia can be a valid alternative to general anesthesia in patients who are undergoing PCNL and can be useful in high-risk patients.^1^ In our case, general anesthesia with a laryngeal mask was performed without difficulties.

The patient position is a key point, because in this group of patients the access area is limited, and even sometimes getting the proper position can be a challenge in itself.^4^ Lateral decubitus may be an easier alternative.^2^

The percutaneous access can be made under ultrasonographic, fluoroscopic, or CT control. These complex situations may necessitate the combination of these techniques. CT is the imaging modality of choice for detecting renal calculi and also has the possibility of three-dimensional reconstruction to delineate the collecting system. This makes this technique the most versatile and sensitive imaging modality for preoperative and postoperative evaluation. Despite all these precautions, it should be noted that in this group of patients, visceral injury during the puncture of the renal cavities is one of the major risks to be considered, because of its anatomical abnormalities.^1^

## Conclusion

The treatment of renal stones in patients with spinal deformities is a complex situation, which may necessitate different approaches in planning and performing percutaneous procedures. Special attention to respiratory function is necessary to avoid complications. These cases represent a challenging but a feasible situation.

## Abbreviations Used

CT: computed tomography
PCNL: percutaneous nephrolithotomy
KUB: kidney, ureter, and bladder radiography
